# Supplementary material for: Using a Fibrinolysis Delivery Catheter in Pulmonary Embolism Treatment for Measurement of Pulmonary Artery Hemodynamics
Source: Adv Respir Med. 2022 Nov 23;90(6):483–99. doi: 10.3390/arm90060055 (PMC9774279; doi:10.3390/arm90060055)
Supplement: Supplementary file 1 [file arm-90-00055-s001.zip › arm-1969448-supplementary.pdf]

SUPPLEMENTARY MATERIAL

- The original figures of invasive right and left pulmonary artery pressure measurements at catheter laboratory and at intensive care unit using EKOS drug delivery catheter are redesigned using DigitizeIt program version 2.5.10.

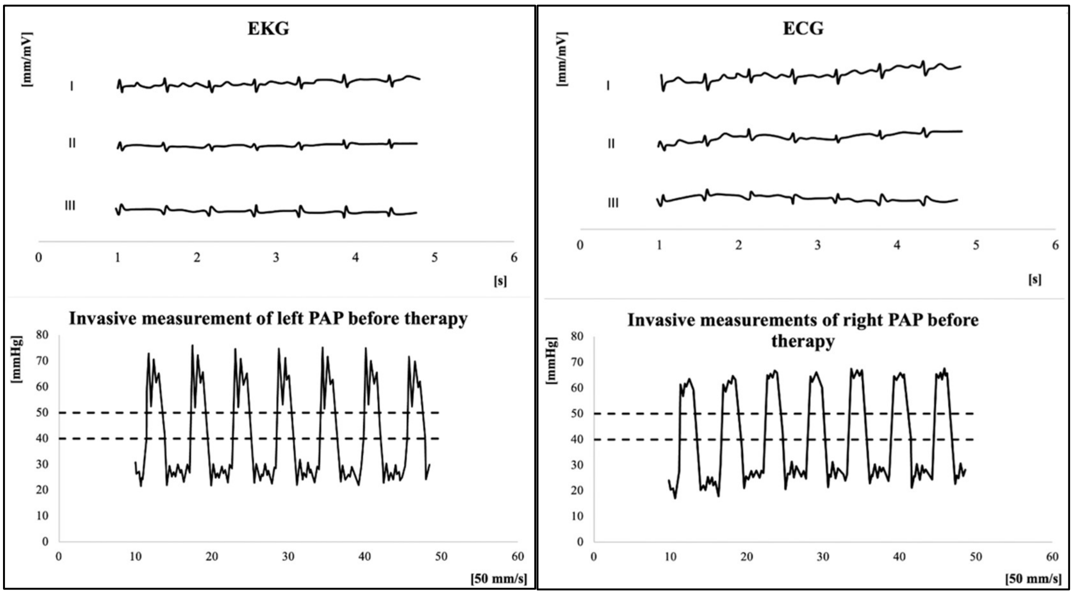

Figure S1:Right and left pulmonary artery pressure measurement respectively before therapy initiation

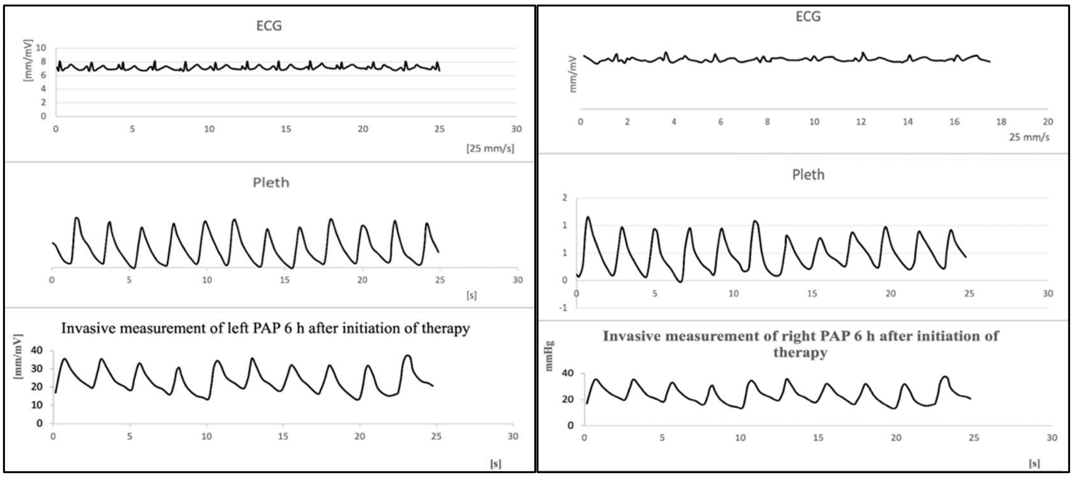

Figure S2: Right and left pulmonary artery pressure measurement respectively 6h after therapy initiation at ICU.
